# Detecting and supporting COVID-19 outbreak response in a small-scale nursing home through passive wastewater surveillance of SARS-CoV-2

**DOI:** 10.1128/aem.00757-26

**Published:** 2026-07-23

**Authors:** Min Ki Jeon, Doris Yoong Wen Di, Maria Steadmon, Stella Maris Remigio, Pamela O'Brien, Daniel P. Strange, Vila Chanthasouvanh, Dayna Ornellas, Edward Desmond, Tao Yan

**Affiliations:** 1 Water Resources Research Center, University of Hawai‘i at Mānoa, Honolulu, Hawaii, USA; 2 Department of Civil, Environmental and Construction Engineering, University of Hawai‘i at Mānoa, Honolulu, Hawaii, USA; 3 State Laboratories Division, State of Hawai‘i Department of Health, Pearl City, Hawaii, USA; 4 Disease Outbreak Control Division, State of Hawai‘i Department of Health 11147, Honolulu, Hawaii, USA; Centers for Disease Control and Prevention, Atlanta, Georgia, USA

**Keywords:** passive wastewater sampling, respiratory virus, nursing home, small-scale, sequencing

## Abstract

Passive wastewater sampling has emerged as a cost-effective approach for pathogen monitoring, but its application in very small populations has not been well studied. This study reports the use of tampons as passive samplers in a small-scale nursing home of 24 residents to detect respiratory viruses, including SARS-CoV-2, influenza A, influenza B, and respiratory syncytial virus (RSV). Weekly wastewater surveillance by reverse transcription digital PCR (RT-dPCR) for a 1-year period resulted in a cluster of consecutive SARS-CoV-2 detections over 4 weeks. When wastewater SARS-CoV-2 concentrations reached higher levels in the third and fourth weeks, whole-genome sequencing achieved >98% genome coverage, identifying lineage LB.1.5, while lower-concentration samples produced only partial genomes. Clinical testing was initiated in the fourth week when clinical symptoms also emerged, and confirmed SARS-CoV-2 infection in 15 of 24 residents and staff. Sequencing of the clinical samples confirmed LB.1.5 in 11 cases, corroborating the wastewater findings. Influenza A subtypes H3N2 and H1N1 were additionally detected in wastewater by targeted enrichment sequencing, and RSV was detected once by RT-dPCR, neither of which was associated with confirmed clinical cases. These results demonstrate that wastewater surveillance can precede clinical symptoms for early detection, and passive sampling using tampons is a practical and sensitive strategy for outbreak surveillance in small-scale facilities. The study also highlights the need for clear response protocols that include rapid clinical testing and cohorting measures when wastewater viral concentrations exceed defined thresholds.

**IMPORTANCE:**

Nursing homes are among the highest-risk settings for infectious disease outbreaks, yet early detection remains challenging due to the high prevalence of asymptomatic infections and limited resources for routine clinical surveillance. This study demonstrates that inexpensive tampon-based passive wastewater samplers can detect SARS-CoV-2 up to 3 weeks before clinical symptoms emerge in a nursing home with only 24 residents, which is the smallest population in which this method has been validated. Wastewater-derived genomes matched clinical sequencing quality, enabling identification of a novel SARS-CoV-2 lineage new to Hawai'i. These findings support passive wastewater surveillance as a practical early warning tool for small congregate care facilities, provided that clear institutional response protocols are in place.

## INTRODUCTION

Wastewater surveillance has emerged as a powerful tool for monitoring infectious diseases at the community level, providing early warning signs of outbreaks before clinical cases are reported (1–3). Traditional wastewater sampling methods, including grab and composite sampling, have been widely used to detect severe acute respiratory syndrome coronavirus 2 (SARS-CoV-2) and other pathogens, but these methods require specialized equipment, frequent sample collection, and trained personnel, making them costly and logistically demanding (4). Passive sampling has gained increasing attention as an alternative approach that involves placing absorbent materials, including cotton gauze (5–7), cotton cheesecloth (8), electronegative membranes (9), and polymer membranes (10), into wastewater systems to passively accumulate viral and bacterial targets over time. This method eliminates the need for complex instrumentation or interface settings and hence can be deployed in a wide range of environments, including locations where autosamplers are impractical due to limited space, lack of power supply, or the difficulty of transporting equipment (11). By passively and continuously accumulating viral particles over time, passive sampling increases the likelihood of detecting low-level viral shedding from individual building-level sewage (12) and provides a more comprehensive view of viral circulation (13).

Although studies have demonstrated the feasibility of tampon-based passive sampling across a range of building sizes (12, 14), most studies have focused on facilities with large populations, with the smallest previously validated population being 86 residents (14). Whether or not this approach is effective for smaller population settings, defined here as residential facilities with fewer than 30 residents, requires additional testing, because the small contributing population and the varying individual viral discharge patterns together can result in more viral loading sporadicity with decreased detection sensitivity (15). Nursing homes are excellent examples of such small population settings, because of the vulnerable populations and heightened risk of severe diseases from outbreaks (16). Routine clinical testing can be procedurally challenging due to staff resource constraints and sampling consent requirements and even unproductive because of the often asymptomatic infections among the population (17, 18). For nursing homes, passive sampling offers a non-invasive and cost-effective solution that can potentially detect viral circulation before outbreaks occur (11).

In this study, we evaluated the effectiveness of tampon-based passive sampling for wastewater surveillance in a small nursing home with a total population of 24 residents, which is approximately three times smaller than the smallest previously published application of this method (14) to detect several important respiratory viruses, including SARS-CoV-2, influenza A and B viruses, and respiratory syncytial virus (RSV). Clinical testing was initiated following the continuous detection of SARS-CoV-2 in wastewater for 4 weeks, prompting a rapid response by nursing home staff to isolate infected individuals and manage symptoms. Whole-genome sequencing (WGS) of clinical samples and hybridization-based targeted enrichment sequencing of wastewater samples were performed to determine whether the same SARS-CoV-2 variant was present in both types of samples and to detect other co-circulating respiratory viruses.

## RESULTS

### SARS-CoV-2 detection in wastewater through tampon sampling

**Fig 1:**
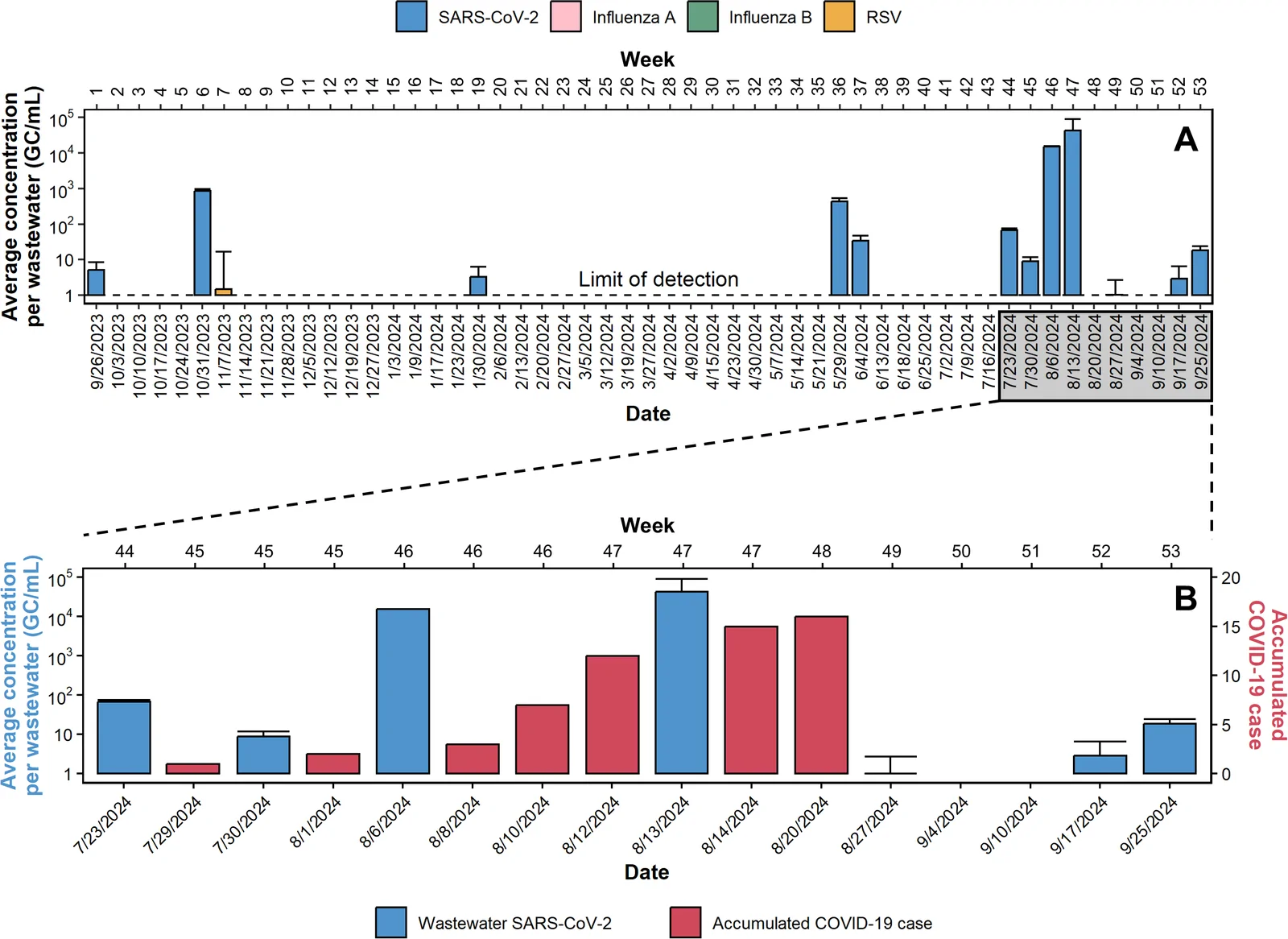
(**A**) Detection of four targeted respiratory viruses (SARS-CoV-2 [blue], influenza A [pink], influenza B [green], and RSV [yellow]) in nursing home wastewater using tampons as passive samplers. (**B**) Timeline of accumulated COVID-19 clinical cases (red) and wastewater detections of SARS-CoV-2 (blue) during the COVID-19 outbreak in the nursing home. The upper *x*-axis shows sampling weeks, and the lower *x*-axis shows sampling dates. Error bars represent standard deviation (*n* of wastewater samples replicates tested per sampling event = 4).

RNA samples extracted from the passive wastewater sampler tampons were tested using RT-dPCR to detect and quantify SARS-CoV-2, influenza A, influenza B, and RSV (Fig. 1A). Positive SARS-CoV-2 signals were detected from wastewater in Weeks 1, 6, 19, 36, and 37; however, no clinical COVID-19 cases were reported from the nursing home during these periods. Between Weeks 44 and 47, SARS-CoV-2 RNA was consistently detected over a consecutive 4-week period for the first time using this method. During the first 2 weeks of this period (Weeks 44 and 45), average SARS-CoV-2 RNA concentrations in wastewater were relatively low, measuring 67.8 ± 7.5 GC/mL and 8.9 ± 2.8 GC/mL, respectively. However, significantly higher average concentrations were observed starting from Week 46 and peaking at Week 47, reaching 1.5 × 10^4^ ± 2.1 × 10^2^ GC/mL and 4.3 × 10^4^ ± 4.6 × 10^4^ GC/mL, respectively. On 8/27/2024, only one of four total biological/technical replicates tested positive with an average of 0.9 ± 1.8 GC/mL, which was below the 1 GC/mL detection limit. After a month, SARS-CoV-2 RNA signals were detected again from Weeks 52 to 53, with low average concentrations measuring 2.9 ± 3.6 GC/mL and 18.5 ± 5.6 GC/mL, respectively, but with no COVID-19 cases reported. The quantified SARS-CoV-2 RNA concentrations were used to determine input volumes for downstream hybridization-based target enrichment sequencing. No influenza A or B viruses were detected in the wastewater samples throughout the surveillance period. RSV was detected in a sample collected in Week 7 with a low concentration (1.5 ± 15.4 GC/mL); however, no corresponding clinical cases were reported at the facility. As a result, SARS-CoV-2 became the primary focus of this study.

### COVID-19 infection of nursing home residents and actions

Following the detection of SARS-CoV-2 RNA in wastewater sampled in Week 44 (7/23/2024), the wastewater surveillance team immediately notified the nursing home facility administrator upon receiving the RT-dPCR results on 7/26/2024 within the same week. On 7/29/2024 (Week 45), the facility administrator reported that although one staff member had been out with COVID-19 during the previous week, no residents exhibited respiratory symptoms or tested positive using at-home rapid antigen tests. On 8/1/2024 (Week 45), a newly hired staff member (an activities assistant), who began working from 7/29/2024 (Week 45), had tested positive for COVID-19 and remained at home until testing negative. When the wastewater surveillance team reported the wastewater RT-dPCR results from the 8/6/2024 (Week 46) sample (reported on 8/8/2024 (Week 46)), showing a more than 1,700-fold increase in SARS-CoV-2 RNA concentration compared to earlier samples, no staff or residents had tested positive or shown symptoms of COVID-19. The activities assistant had tested negative and returned to work on 8/6/2024 (Week 46).

One family member of a nursing home resident, who visited the facility daily from 7/28/2024 to 8/1/2024 (both in Week 45), tested positive for COVID-19 on 8/8/2024 (Week 46) using an at-home rapid antigen testing kit. Six residents were confirmed positive for COVID-19 following tests conducted on the evening of 8/10/2024 (Week 46), and five additional positives were confirmed on 8/12/2024 (Week 47). These residents began Paxlovid treatment and were placed in isolation. In response, the facility implemented additional mitigation measures: remaining residents were grouped into smaller cohorts for activities and meals, family members and visitors were advised to limit non-essential visits, enhanced cleaning protocols were initiated, and new admissions were temporarily suspended. At that time, no staff members exhibited respiratory symptoms or tested positive for COVID-19, and they were equipped with full personal protective equipment (PPE), including N95 respirators, gowns, gloves, and face shields. The number of positive cases increased over time, with a total of 14 residents testing positive on 8/14/2024 (Week 47), followed by one additional case on 8/20/2024 (Week 48) (Fig. 1B). All positive COVID-19 test results from the nursing home residents and staff were confirmed using at-home rapid antigen tests. No further resident cases were reported after that date. A detailed timeline of confirmed COVID-19 cases and key infection control actions is provided in Table S3.

### Clinical SARS-CoV-2 test result

A total of 24 NP swab samples were screened for SARS-CoV-2 from nursing home residents using RT-qPCR, with 15 samples testing positive for both N and ORF genes. All 15 positive cases were from nursing home residents, with an average age of 89.0 ± 8.9, and no staff members tested positive. Cycle threshold (Ct) values for the N gene ranged from 14.07 to 30.90 (average: 22.95 ± 4.53), while those for the ORF gene ranged from 14.01 to 32.01 (average: 22.63 ± 4.76). A linear regression analysis between the Ct values of the N and ORF genes yielded a slope of 0.93, a *y*-intercept of 1.85, and an *r*² value of 0.96, indicating a strong correlation between the two targets (Fig. S2). One sample (C14), which had the highest Ct values for both N and ORF genes, was not sequenced due to low concentration. As a result, 14 samples were subjected to full-length SARS-CoV-2 WGS.

### Comparison between wastewater and clinical sequencing results

**Fig 2:**
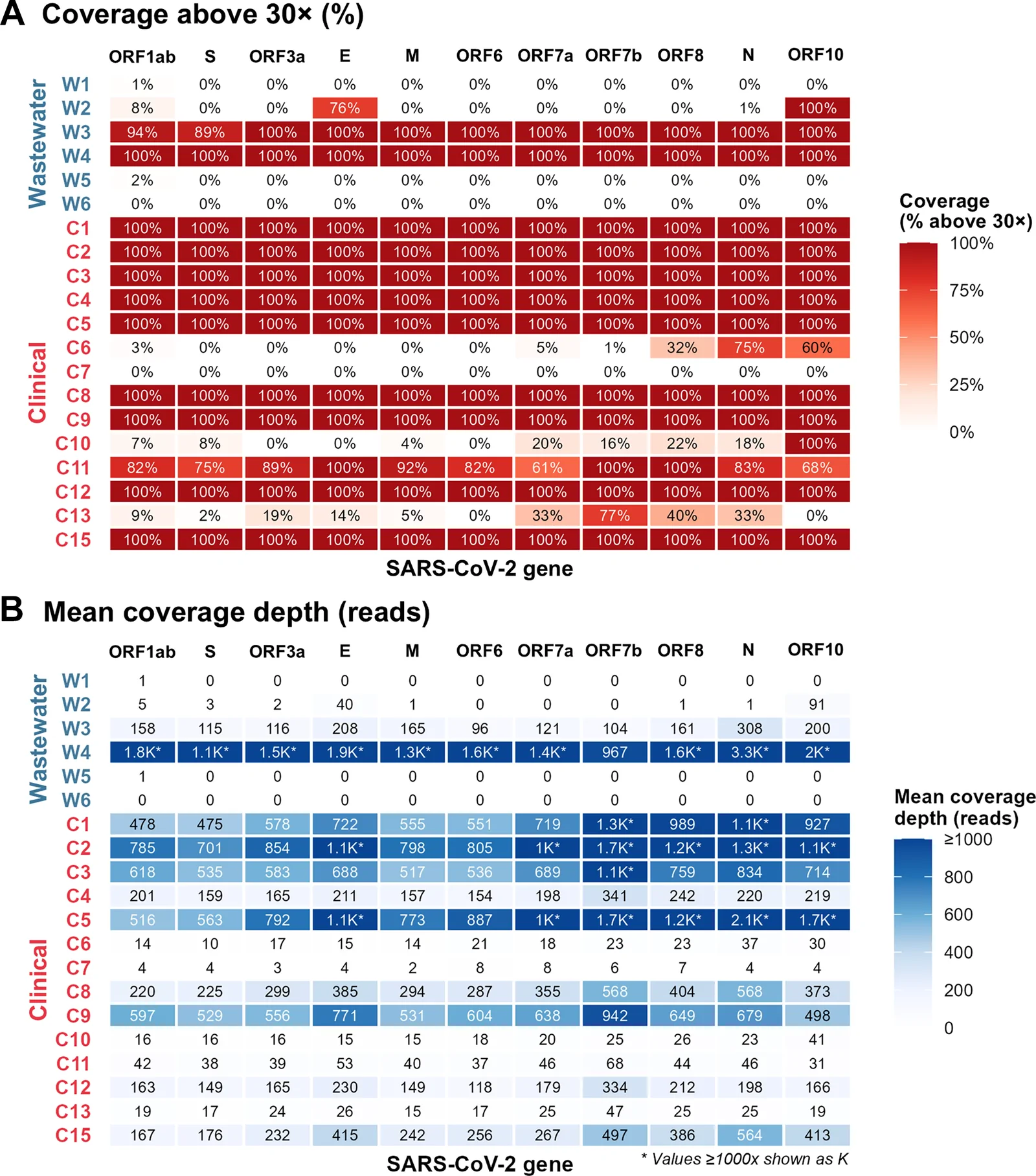
Per-gene SARS-CoV-2 sequencing coverage comparison between wastewater and clinical samples. (**A**) Percentage of each gene covered above 30× sequencing depth for wastewater samples W1–W6 and clinical samples C1–C15. (**B**) Mean sequencing coverage depth (reads) per gene for the same samples. Wastewater samples are labeled as blue text, and clinical samples are labeled as red text. Genes are ordered by genomic position from left to right. Values ≥1,000× in panel b are shown as K. Asterisks (*) denote values truncated for visualization. Samples W3 and W4 were collected during the peak of the COVID-19 outbreak (weeks 3 and 4), while W1, W2, W5, and W6 correspond to periods of low viral concentration.

Assembled SARS-CoV-2 consensus genomes from both wastewater and clinical samples with coverage greater than 76.0% were consistently assigned to the same Pango lineage, LB.1.5 (Table 1; Table S2). In contrast, samples with coverage below 21.3% could not be reliably classified, as lineage assignments would have relied on only small fragments of the viral genome. This variation in sequencing success reflected substantial differences in viral load at the time of collection (Fig. 2), where high coverage levels directly correlated with elevated SARS-CoV-2 concentrations and strong N or ORF gene signals in both sample types. In wastewater samples, SARS-CoV-2 sequencing quality improved significantly from Weeks 44 to 47 (Fig. 2; Fig. S3), as coverage increased and the number of unidentified nucleotides (Ns) decreased, likely due to a rise in infection levels within the nursing home. Similarly, the majority of the clinical samples (10 out of 14) yielded high genome coverage, likely due to their collection timing coinciding with peak SARS-CoV-2 concentrations in wastewater.

**TABLE 1:** Whole-genome sequencing results of SARS-CoV-2 genomes from wastewater and clinical samples

Sample type	Sample ID	Sampling date	Closest clade	Closest Pango lineage	WHO^*a*^ name	No. of Ns	Total aligned target read counts	% Coverage (reference length: 29,903 bp)	Avg SARS-CoV-2 concentration^*b*^ (GC/mL for wastewater samples; Ct value for clinical samples)	
									N gene	ORF gene
Wastewater	W1	7/23/2024	21K	BA.1.1	Omicron	28,815	278	3.2%	57.6	–^*d*^
	W2	7/30/2024	24C	MK.1	Omicron	23,379	2,115	21.3%	7.5	–
	W3	8/6/2024	24A	LB.1.5	Omicron	475	47,884	97.9%	1.3 × 10^4^	–
	W4	8/13/2024	24A	LB.1.5	Omicron	21	554,563	99.5%	3.6 × 10^4^	–
	W5	9/17/2024	Insufficient depth for confident calling**^*c*^**						3	–
	W6	9/25/2024	Insufficient depth for confident calling						15.4	–
Clinical	C1	8/14/2024	24A	LB.1.5	Omicron	0	159,008	99.3%	14.07	14.01
	C2		24A	LB.1.5	Omicron	60	245,277	99.3%	17.73	16.4
	C3		24A	LB.1.5	Omicron	60	187,113	99.3%	17.78	16.41
	C4		24A	LB.1.5	Omicron	70	59,085	99.3%	21.16	19.89
	C5		24A	LB.1.5	Omicron	60	192,165	99.3%	19.94	20.89
	C6		24A	LB.1.5	Omicron	7,038	4,649	76.0%	24.34	24.66
	C7		24G	KP.2.3	Omicron	26,129	1,434	12.2%	28.69	26.65
	C8		24A	LB.1.5	Omicron	60	73,480	99.3%	22.93	23.48
	C9		24A	LB.1.5	Omicron	60	175,108	99.3%	21.83	20.83
	C10		24A	LB.1.5	Omicron	1,568	5,398	94.3%	26.83	26.79
	C11		24A	LB.1.5	Omicron	211	12,695	98.8%	25.82	25.22
	C12		24A	LB.1.5	Omicron	77	48,924	99.3%	23.99	23.41
	C13		24A	LB.1.5	Omicron	740	6,287	97.0%	26.94	26.87
	C14		Not sequenced due to low concentration						30.9	32.01
	C15	8/20/2024	24A	LB.1.5	Omicron	70	59,475	99.3%	21.35	21.92

^*a*^ World Health Organization.

^*b*^ Quantification methods: RT-dPCR for wastewater samples; RT-qPCR for clinical samples.

^*c*^ Only partial detection in the ORF1ab.

^*d*^ Not applicable as the detection kit used for wastewater samples did not include the *ORF* gene.

Building on these concentration-driven trends, sequencing coverage varied across all samples, mirroring the fluctuations in viral load (Fig. 2). Among wastewater samples, W3 and W4 achieved sufficient coverage for reliable genomic characterization, with ORF1ab coverage of 93.95% and 100%, and mean sequencing depths of 157.59× and 1,806.73×, respectively. Conversely, samples W1, W2, W5, and W6 yielded near-zero coverage across all genes, consistent with their low RT-dPCR concentrations. Clinical samples followed a similar pattern: 10 of 14 (C1–C5, C8–C9, C11–C12, and C15) achieved ≥80% genome coverage with mean depths ranging from 163.49× to 784.97×, while C6 and C7 showed markedly lower coverage (2.77% and 0%, respectively), indicating insufficient viral RNA for complete recovery. Notably, the per-gene coverage profiles of wastewater samples W3 and W4 were comparable to those of the high-quality clinical samples, demonstrating the reliability of wastewater-based genomic characterization during periods of high infection.

**Fig 3:**
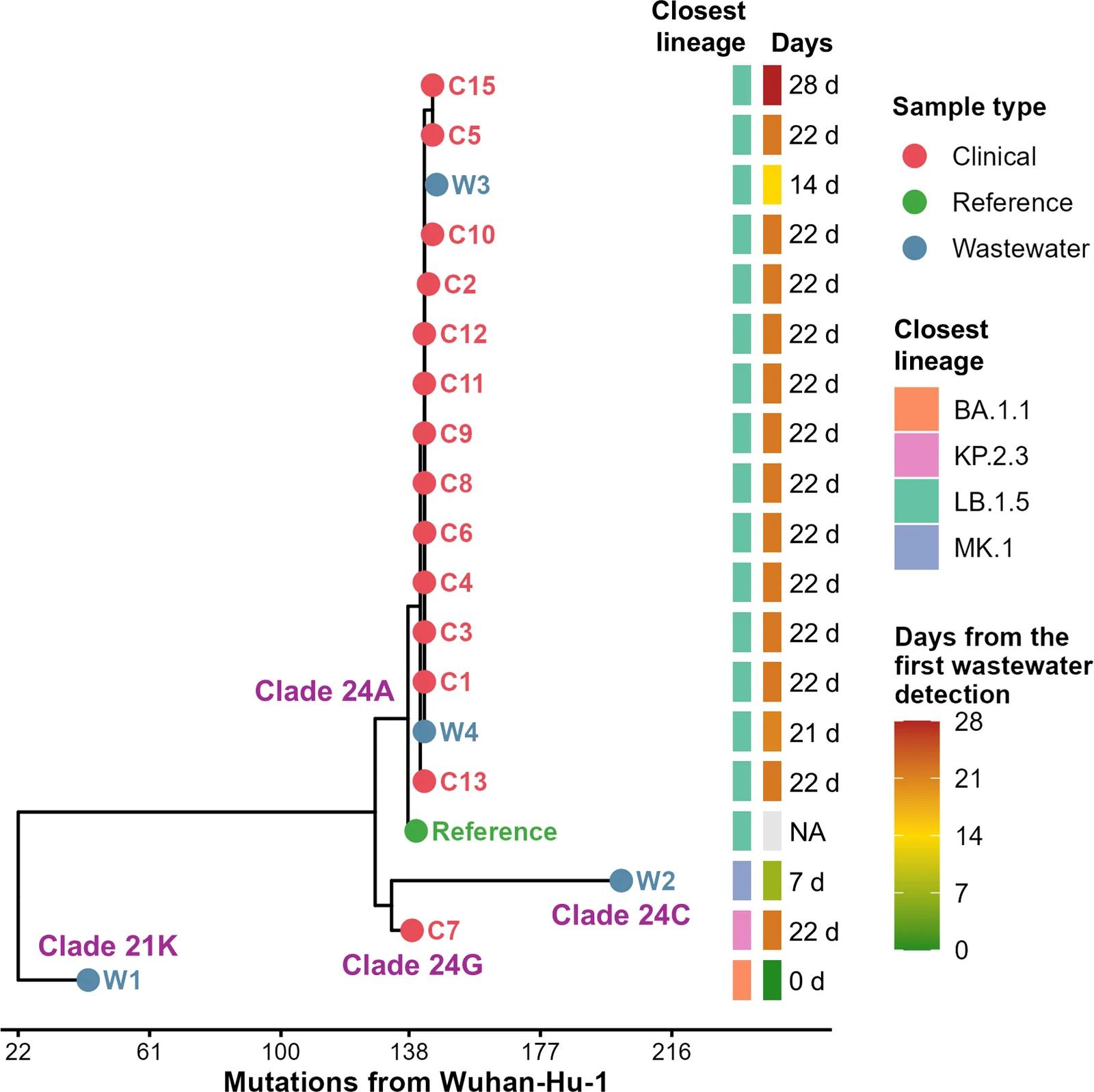
Phylogenetic analysis of SARS-CoV-2 consensus sequences from wastewater and clinical samples collected during a nursing home outbreak. A maximum likelihood phylogenetic tree was constructed from SARS-CoV-2 consensus sequences obtained from wastewater samples (W1–W6; blue dots), clinical nasopharyngeal swab samples (C1–C15; red dots), and a reference genome (green dot). The LB.1.5 reference genome (SARS-CoV-2/human/USA/CA-CDPH-500140229/2024; GenBank accession: PQ134785.1) was included for lineage comparison. Colored-annotation strips to the right of the tree indicate the closest SARS-CoV-2 lineage assigned by Nextclade (left strip) and the number of days elapsed from the first wastewater detection of SARS-CoV-2 on 7/23/2024 (right strip). SARS-CoV-2 clade designation is shown as purple text on the tree. Wastewater samples W5 and W6 were excluded from the tree due to low assembled sequences.

Phylogenetic tree analysis revealed that high-coverage SARS-CoV-2 consensus genomes from both wastewater and clinical samples were highly related, clustering within clade 24A and lineage LB.1.5, with minimal amino acid mutational differences ranging from 0 to 4 between the sequences (Fig. 3). These sequences were most closely related to the reference genome used for the phylogenetic tree, lineage LB.1.5 (SARS-CoV-2/human/USA/CA-CDPH-500140229/2024, GenBank accession: PQ134785.1), with the exception of homoplastic mutational differences at C25782T and C26885T. Amino acid differences between SARS-CoV-2 consensus sequences from wastewater and clinical samples were minimal, at most one mutation per position, which may be attributed to either sequencing errors or mutations that occurred during viral replication within hosts (Fig. 3). Wastewater samples W5 and W6 were excluded from the phylogenetic tree construction due to low assembled sequences for SARS-CoV-2.

**Fig 4:**
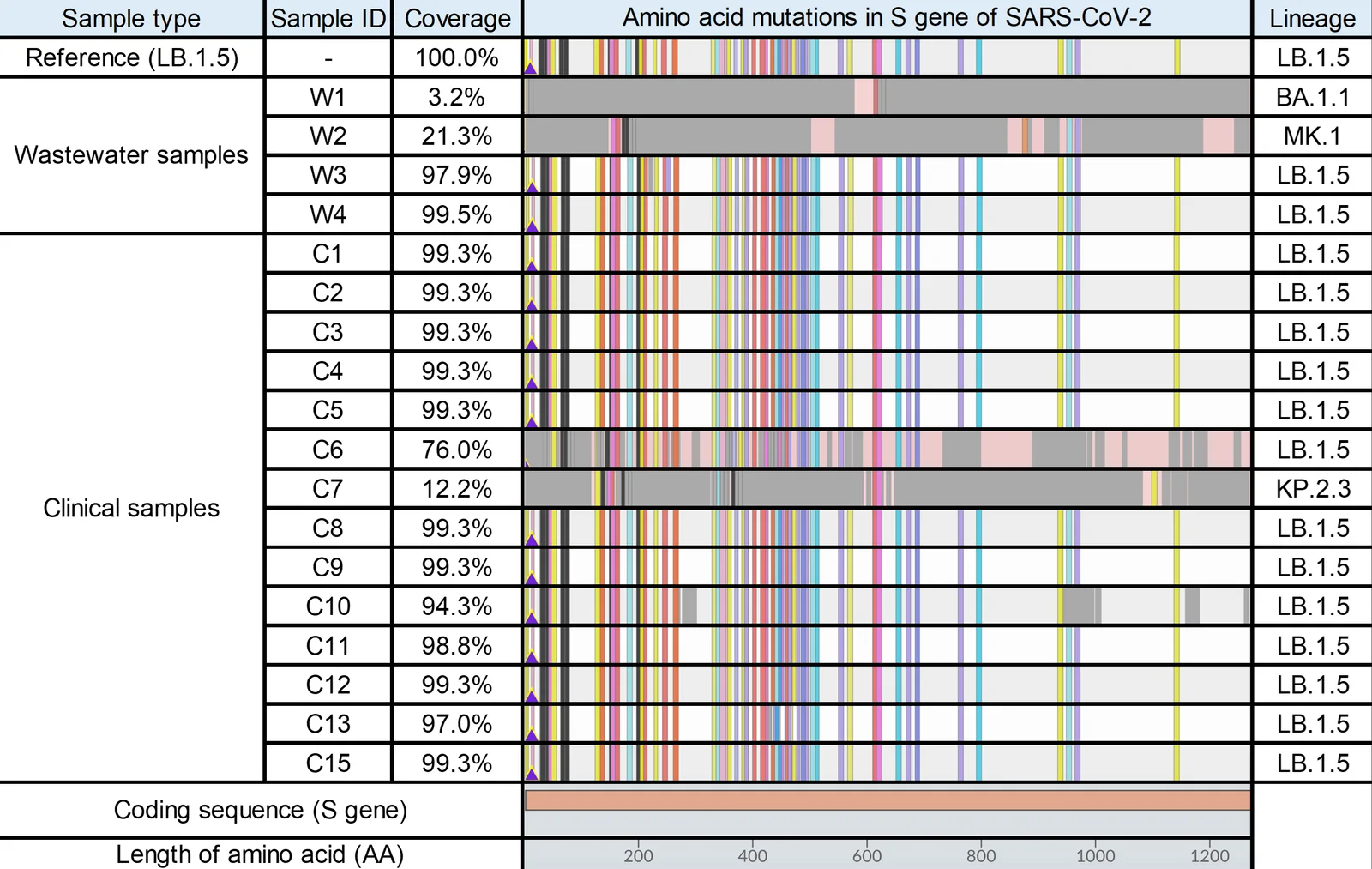
Amino acid mutations in the SARS-CoV-2 spike (S) protein gene from consensus sequences of wastewater, clinical samples, and the LB.1.5 reference genome SARS-CoV-2/human/USA/CA-CDPH-500143070/2024 (GenBank accession: PQ575878.1). Colored lines indicate amino acid mutations at each corresponding position compared to the SARS-CoV-2 reference genome Wuhan-Hu-1/2019 (GenBank accession: NC_045512.2). Gray regions represent missing sequence data, while red regions indicate assembled sequences from samples with genome coverage below 80%.

Closer examination of mutational patterns in the S gene, which contains the highest density of mutations in the SARS-CoV-2 genome, revealed a consistent trend across the reference genome, wastewater, and clinical samples. Samples with low genome coverage (W1, W2, and C7) only yielded less than 21.3% of the partial S gene sequences, which limited confident clade and Pango lineage classification. However, some mutations observed in these low-coverage samples aligned with those identified in higher-coverage consensus genomes (Fig. 4). For example, wastewater sample W2 contained the mutations F157S and R158G in the S gene, which was first observed in clade 23I, the ancestral lineage of clade 24A (Fig. 3). Clinical samples C6 and C10 also exhibited low genome coverage yet yielded sufficient mutational markers to assign both clade and Pango lineage classifications. High-coverage sequences from both wastewater and clinical samples showed no significant amino acid differences in the S gene. Minor variations observed among these sequences may have resulted from sequencing errors, natural mutations during viral replication, or RNA degradation, though the exact cause could not be determined.

### Other respiratory viruses

Beyond SARS-CoV-2, targeted enrichment sequencing revealed co-circulation of additional respiratory viruses during the surveillance period, which were not detected from RT-dPCR. Influenza A virus subtypes H3N2 and H1N1 were detected in wastewater samples W2 and W4, respectively, using the targeted enrichment sequencing (Table S2). Subtype assignment was based on the simultaneous detection of partial sequences from both the hemagglutinin (HA) and neuraminidase (NA) gene segments within the same sample using the DRAGEN Microbial Enrichment Plus pipeline, which performs multi-segment classification. The co-detection of HA (H3 in W2 and H1 in W4) and NA (N2 in W2 and N1 in W4) segments within the same sample enabled subtype linkage. These detections were supported by low read counts of 353 reads for H3N2 and 252 reads for H1N1, with corresponding genome coverages of 6.7% and 5.8%, respectively. In sample W2, partial H3N2 sequences were identified across multiple genomic regions, including basic polymerase 2 (PB2; 11.9% coverage, 16 aligned reads), HA (21.8%, 161 reads), nucleocapsid protein (NP; 4.1%, 64 reads), NA (2.9%, 22 reads), and matrix protein (M; 13.8%, 90 reads). In sample W4, partial sequences of H1N1 were recovered from PB2 (6.6%, 56 reads), polymerase acidic (PA; 22.4%, 166 reads), NP (5.5%, 18 reads), and nuclear export protein/nonstructural protein 1 (NEP/NS1; 5.3%, 12 reads). No additional respiratory viruses were detected in the wastewater samples based on the wastewater sequencing results.

## DISCUSSION

While the use of tampons as passive samplers for building-level wastewater surveillance has been previously demonstrated in larger populations ranging from 86 to several thousand individuals (12, 14, 19, 20), the applicability of this approach in much smaller populations has not been reported before. Our study provides valuable evidence that passive sampling using tampons is feasible and effective, even in a small nursing home with only 24 residents. Additionally, SARS-CoV-2 detection was successful with a small sample volume of wastewater squeezed from the tampons (10 mL) that was sampled only once per week. Despite the small population size and presumably low initial viral load, SARS-CoV-2 was consistently detected, with concentration gradually increasing over the 4 weeks leading up to the onset of clinical symptoms and laboratory-confirmed cases. Wastewater sequencing efforts became successful during the later weeks (W3 and W4 samples) of surveillance, corresponding with higher viral concentrations in the samples. These findings suggest that even with a small volume of samples and infrequent sampling, it can provide early and reliable signals of SARS-CoV-2 circulation.

Two key factors that likely contributed significantly to the success of passive wastewater surveillance in a small nursing home would be (i) the use of nanoparticles for efficient viral concentration and (ii) the targeted library enrichment for respiratory virus sequencing. The concentration of targeted respiratory viruses in wastewater is relatively low compared to background materials such as human cellular debris, bacteria, and bacteriophages, which are commonly abundant in wastewater (21). The combined use of nanoparticles and the targeted library enrichment method can effectively reduce the proportion of non-target organisms and background genetic material in wastewater, increasing the likelihood of detecting and characterizing low concentrations of respiratory viruses from a small-scale nursing home.

Targeted enrichment sequencing successfully recovered SARS-CoV-2 genomes from samples with higher viral concentrations; however, genome recovery was not achieved in all RT-dPCR-positive samples. For instance, samples W5 and W6, despite testing positive by RT-dPCR, yielded no sequencing data, and samples W1 and W2 produced only low genome coverages. These findings are consistent with previous reports that samples with SARS-CoV-2 Ct values above 32.4 produced genome coverages as low as 55.2%, compared to near-complete coverage (~99.8%) for samples with lower Ct values (22). This suggests that while current enrichment strategies improve detection, further optimization of hybridization probe design or library amplification protocols may be necessary to enhance sequencing sensitivity for low-viral-load wastewater samples.

During the peak of infection, wastewater samples W3 and W4 and the majority of clinical samples (10 out of 14) showed sufficiently high sequencing coverage above 30× and quality to support reliable genomic comparison (Fig. 2 and 3). All high-coverage clinical genomes were phylogenetically highly related, and the outbreak most likely originated from a single introduction event prior to the branch point at mutation 138 (Fig. 3), consistent with a single-source outbreak. Although transmission pathways could not be confirmed without formal contact tracing, the epidemiological context suggests that the new staff member who worked from 7/29/2024 (Week 45) or the visitor who made daily visits between 7/28/2024 and 8/1/2024 (both within Week 45) may have been the index case. Wastewater sample W4 showed 99.5% genome coverage with no mutational differences from clinical samples, while W3, collected 1 week earlier, carried a unique S gene mutation (T250K), placing it on a separate but closely related phylogenetic node. This pattern was also observed among clinical samples, consistent with natural intra-host evolution within a single outbreak rather than an unrelated source (23), supporting the conclusion that the wastewater and clinical detections are linked. The LB.1.5 lineage detected in this outbreak is a descendant of LB.1, which was among the predominant SARS-CoV-2 lineages circulating nationally during the summer of 2024, reaching greater than 10% prevalence in the United States by late May 2024 (24). However, a review of the Nextclade database and NCBI Virus records by the Hawai'i Department of Health confirmed that no sequences matching this specific LB.1.5 lineage had been previously reported from Hawai'i at the time of the outbreak, making these the first documented cases of LB.1.5 in the state (Fig. 2). This suggests that while the lineage was consistent with national circulation patterns, it represented a novel introduction into the geographically isolated Hawai'i community, underscoring the value of building-level genomic wastewater surveillance for tracking variant introductions at the local level.

While this study demonstrates the successful early detection of infections using tampon-based passive wastewater sampling in a small nursing home, it also highlights important considerations for translating wastewater signals into timely public health responses in similar settings. In this facility, wastewater surveillance data were communicated to both the nursing home staff and epidemiologists on a weekly basis, and the facility was responded to by maintaining heightened monitoring of residents and reinforcing COVID-19 protocols following initial wastewater detections. However, additional elevated infection control measures were formally initiated only after the number of confirmed COVID-19 cases among residents rose (Table S3), suggesting that clinical case counts ultimately served as the primary trigger for escalating the response rather than the wastewater signal alone. Although rapid antigen tests continued to yield negative results in the early weeks of detection, consistent with their well-documented limitations in detecting low viral loads during early or asymptomatic infection (25, 26), the wastewater data alone were not sufficient to prompt the full escalation of infection control measures in the absence of confirmed clinical cases. This sequence raises an important question with direct implications for wastewater surveillance programs beyond this facility: would other nursing homes act on a wastewater signal alone, in the absence of clinical symptoms or positive diagnostic tests? Based on our experience, the answer remains uncertain, as the absence of visible illness and negative rapid antigen results created a false sense of reassurance that the wastewater data alone could not overcome. This is not a failure of the surveillance technology itself, but rather reflects the absence of predefined, tiered action protocols that trigger responses based on wastewater signals alone. To bridge this critical infrastructure gap, future implementations in congregate settings must establish clear, independent mandates for action that do not rely on lagging clinical confirmation.

Importantly, wastewater surveillance contributed to a successful outbreak response in which disease transmission was ultimately interrupted. The first COVID-19 case among residents was identified on 8/10/2024 (2 days after the significant increase in wastewater SARS-CoV-2 signal) by rapid antigen testing, and this initial case was asymptomatic, underscoring the value of active monitoring prompted in part by the wastewater data. The last case was identified on 8/20/2024, the same day the resident became symptomatic, following which no further cases were reported. The combination of early wastewater detection, rapid clinical follow-up, and prompt infection control measures (i.e., cohorting, enhanced PPE use, and visitor restrictions) collectively enabled the facility to contain the outbreak within a 3-week window. This outcome demonstrates that tampon-based passive wastewater surveillance, when integrated into a responsive institutional framework, can contribute meaningfully to outbreak containment in small congregate care settings.

This challenge of “unconfirmed” signals is clearly reflected in our one-year surveillance results. Between September 2023 and September 2024, SARS-CoV-2 was detected in wastewater on 12 occasions (Fig. 1A), of which only four were associated with documented COVID-19 cases among residents or staff (Fig. 1B). This pattern reflects the inherent sensitivity of wastewater surveillance, which captures viral signals from asymptomatic, pre-symptomatic, and convalescent individuals; populations that would not typically trigger clinical testing or epidemiological investigation (11, 19). Several factors may explain the remaining eight unconfirmed detections in this small facility. First, convalescent shedding of SARS-CoV-2 RNA in stool is well documented to persist for weeks beyond the resolution of symptoms and the infectious period, and wastewater surveillance cannot reliably distinguish these residual signals from those of new active infections (27). Second, in a facility of only 24 residents, even a single asymptomatic individual shedding at very low levels can generate a detectable wastewater signal, whereas the same level of shedding in a larger population would likely be diluted below the detection threshold (15). Third, non-resident staff members or visitors who do not sleep at the facility could introduce transient viral signals without any corresponding resident illness (28). The low SARS-CoV-2 concentrations measured during these unconfirmed detection events, consistently at or near the 1 GC/mL detection limit, support the interpretation that these signals reflect sporadic, low-level shedding rather than active, sustained transmission within the facility.

Detection outcomes also varied by method. For example, partial influenza A viral fragments were identified in samples W2 and W4 by wastewater-targeted enrichment sequencing but were not detected by RT-dPCR. The detection of partial rather than complete gene sequences in wastewater samples is not uncommon and reflects the degradation of viral RNA in the wastewater matrix, where RNases present in fecal material, microbial enzymatic activity, and cleaning product residues common in healthcare settings fragment RNA molecules into short segments (29). As a result, sequencing coverage across gene segments can be highly uneven, and regions with insufficient coverage are a recognized challenge in wastewater sequencing, especially when the target concentration is low in the sample (30). Although GT Molecular does not publicly disclose the proprietary primer and probe sequences used in the GT-Digital Influenza and SARS-CoV-2 Wastewater Surveillance Assay, the CDC universal assay for influenza A targets the M gene at nucleotide positions 143–226 (31). In contrast, the M gene fragment detected in the wastewater spanned positions 105–193, providing limited overlap with the CDC target region. This incomplete overlap may explain why the sequence was not detected by RT-dPCR. The presence of these subtype-specific gene fragments suggests the circulation of influenza A viruses at low concentrations during the sampling period. Conversely, SARS-CoV-2 was detected in samples W5 and W6 by RT-dPCR but was not recovered through sequencing. These detections did not correspond to or could not be linked to confirmed clinical cases.

Similarly, RSV was once detected in a wastewater sample by RT-dPCR collected on Week 7, but no symptomatic infections were reported at the time. These results showed both the value and limitations of wastewater surveillance, as it has the potential to capture asymptomatic, pre-symptomatic, or undiagnosed infections, but some signals may result from transient viral shedding, resolved infections, or false positives. Without accompanying clinical symptoms or diagnostic screening, such detections cannot be verified. Therefore, the integration of wastewater data with clinical context and testing remains critical for informed public health response and decision-making.

These findings highlight that the effectiveness of wastewater surveillance in congregate settings depends not only on technical performance but also on how facility leadership interprets and responds to surveillance data. Perspectives toward and trust in wastewater data, especially in the absence of confirmed clinical cases, may vary significantly across institutions and shape how, or whether, mitigation strategies are deployed proactively. Also, mandating clinical testing on residents based solely on wastewater data, without clinical symptoms or informed consent, may raise concerns regarding patient rights and medical ethics (32). Thus, future work should consider not only the technical performance of wastewater surveillance but also its integration into facility-level decision-making and public health communication strategies. Ultimately, the success of wastewater surveillance in congregate care settings will depend not only on its technical detection capability but equally on whether facilities have the institutional trust, clear guidance, and predefined response frameworks needed to translate wastewater signals into timely, evidence-based action.

## MATERIALS AND METHODS

### Nursing home selection

The nursing home wastewater surveillance program was a collaborative effort between the wastewater surveillance team from the State Laboratories Division (SLD), State of Hawai‘i Department of Health, and epidemiologists from the Disease Outbreak Control Division, State of Hawai‘i Department of Health. One small nursing home with a total population of 24 residents consented to participate in the program, allowing the collection of weekly wastewater samples by the surveillance team. The facility provides skilled nursing care with a focus on high-acuity residents requiring continuous medical monitoring and assistance with daily living activities. In the event of a suspected outbreak, epidemiologists were granted permission to collect nasal swab samples from residents and staff, and affected residents were placed on High-Risk Respiratory Droplet transmission-based precautions and isolation. Nursing staff caring for these residents are required to use full personal protective equipment (PPE), including gowns, gloves, eye protection, and N95 respirators. Specific details about the facility are withheld to protect the privacy and confidentiality of participants.

### Wastewater passive sampling using tampons

**Fig 5:**
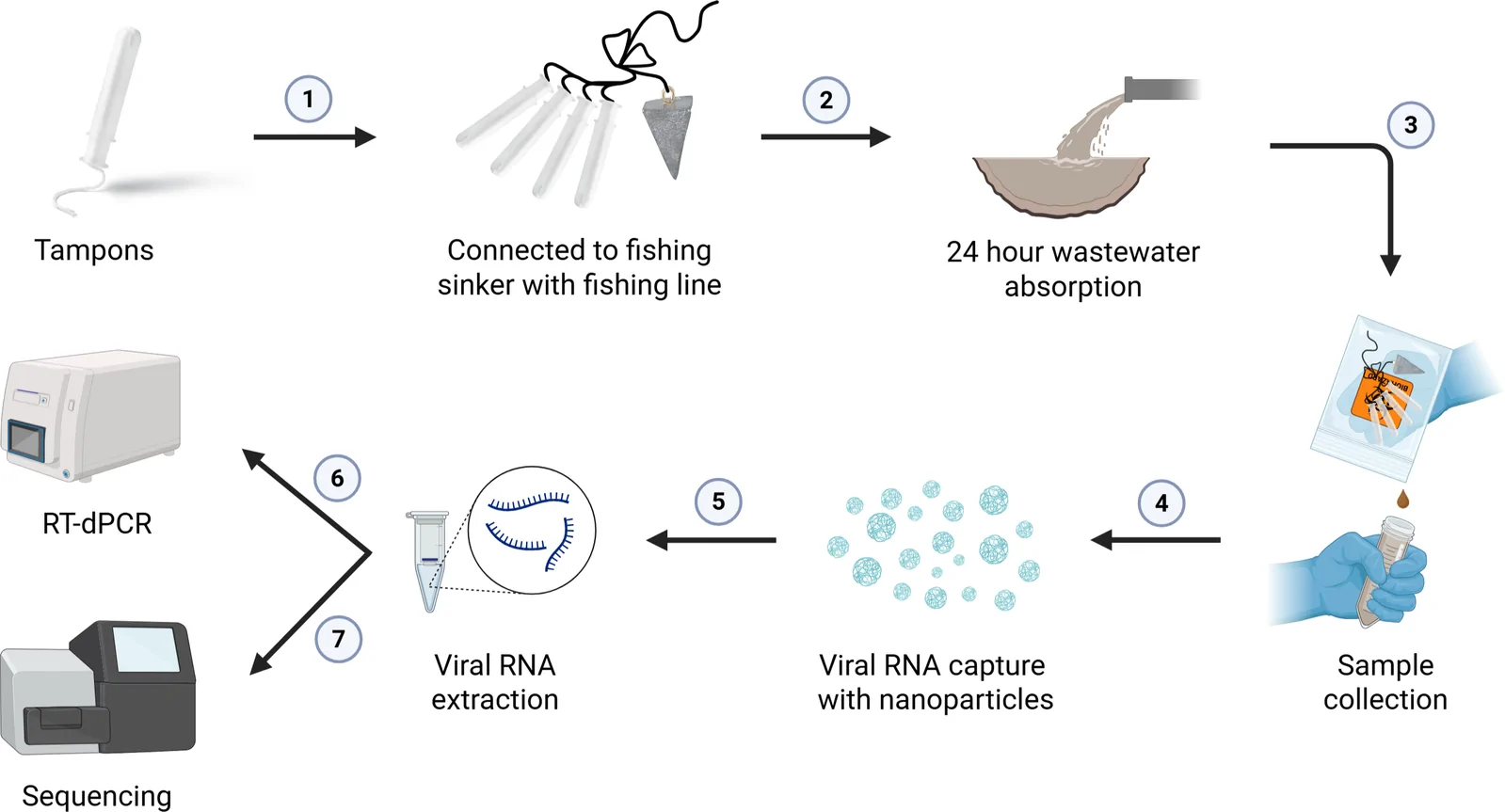
Illustration of the experiment flow of passive wastewater sampling using tampons. Figure created with BioRender.com.

Wastewater sampling was conducted using passive samplers made from Super Plus-size organic tampons (Cora Organic Tampons, Cora, CA, USA) to avoid potential chemical interference. Four tampons were securely tied together using a fishing line and attached to a 5-oz pyramid-shaped plastic-coated steel fishing sinker to ensure they remained submerged and stable against the flow within the sewage pipe (Fig. 5). The assembled sampler was inserted directly into a sewage pipe through a cleanout plug that discharged wastewater from the nursing home. The pipe drains the entire and only the nursing home building, giving us a high level of confidence that the collected wastewater reflects contributions solely from this facility. The fishing line was anchored at the pipe entrance to facilitate the safe deployment and retrieval of the tampons. The passive sampler was inserted into the sewage pipe at 08:00 AM once a week by a wastewater team member from SLD and retrieved after 24 hours starting from 9/26/2023 (Week 1) to 9/25/2024 (Week 53). The sample was placed in a double-sealed, sterilized plastic sample bag, enclosed within a biohazard bag, and transported to the laboratory on ice within one hour of collection. In the laboratory, tampons were manually squeezed inside a biosafety cabinet, yielding approximately 100–150 mL of raw wastewater per sampling event. A total of 40 mL of squeezed raw wastewater samples were filtered through a 200 µm pluriStrainer (PET sieve fabric cell strainer [pluriSelect, Leipzig, Germany]), to remove large particles and debris that could interfere with downstream processing. A total of 10 mL of the resulting liquid fraction per replicate (total of four) was stored in a 15 mL sterilized conical tube and used for RNA concentration and extraction.

### Wastewater viral RNA concentration and extraction

Viral particles were concentrated from the liquid fraction of the squeezed wastewater using Nanotrap Microbiome A Particles (Ceres Nanosciences, VA, USA), which is selective for various viruses, following the manufacturer’s protocol. Bovine coronavirus (BcoV) was spiked into each 10 mL wastewater sample at a concentration ranging from 12,966.6 to 14,820.5 GC/mL as a concentration and extraction control. The BCoV DNA standard was derived from cDNA synthesized via reverse transcription of RNA extracted from a bovine coronavirus vaccine (Zoetis; Kalamazoo, MI, USA). Nanotrap Enhancement Reagent 2 and Nanotrap Microbiome A Particles were added to facilitate viral binding and subsequently resuspended in Nanotrap Buffer 2. The resuspended particles were isolated using a DynaMag-2 magnetic rack (Thermo Fisher Scientific, MA, USA), and viral RNA was extracted using the AllPrep PowerViral DNA/RNA Kit (Qiagen, Hilden, Germany), according to the manufacturer’s instructions. To lyse viral membranes, 500 µL of pre-heated PM-1 buffer (55°C for 10 minutes) from the extraction kit was added to the concentrated viral particles. RNA was then extracted and eluted in 60 µL of DNase/RNase-free water and stored at −80°C until further use. The extracted RNA was used for reverse transcription digital PCR (RT-dPCR) and hybridization-based target enrichment sequencing.

### Quantification of respiratory viruses from wastewater samples

Four RNA respiratory viruses responsible for common respiratory illnesses (SARS-CoV-2, influenza A and B viruses, and RSV) were targeted in wastewater samples. Quantification was performed using the GT-Digital Influenza and SARS-CoV-2 Wastewater Surveillance Assay (Cat# 100305), the GT-Digital RSV Wastewater Surveillance Assay for QIAcuity (Cat# 100360) (GT Molecular, CO, USA), and QIAcuity OneStep Advanced Probe Kit (Qiagen, Hilden, Germany). For SARS-CoV-2, influenza, BcoV, and RSV detection, each 40 µL reaction mixture consisted of 10 µL RNA extracted from the liquid fraction of wastewater, 2 µL of GT-Molecular 20× Flu-SC2-BcoV Assay Master Mix, 2 µL of GT-Molecular 20× RSV Assay, 10 µL of QIAGEN 4× One-Step Viral RT-PCR Master Mix, 0.4 µL of QIAGEN 100× Multiplex Reverse Transcription, and nuclease-free water. For pepper mild mottle virus (PMMoV), which was used as a fecal indicator for wastewater fecal strength and normalization marker, each 40 µL reaction mixture consisted of 1 µL RNA extracted from the liquid fraction of wastewater, 2 µL of GT-Molecular 20× PMMoV Assay, 10 µL of QIAGEN 4× One-Step Viral RT-PCR Master Mix, 0.4 µL of QIAGEN 100× Multiplex Reverse Transcription, and RNase- and DNase-free water. All targets except PMMoV were tested in technical replicates for each biological replicate, while PMMoV was tested only in biological replicates. PMMoV was not detected in the Week 1 sample, likely due to excessive sample dilution prior to method optimization. Following optimization, PMMoV was successfully detected in all subsequent wastewater samples. BcoV was recovered from all samples throughout the study period (Fig. S1). Positive controls for all targets, non-template control (NTC), concentration control, and extraction control were included on each RT-dPCR run.

All reactions were loaded into QIAcuity 26k Nanoplates and processed on the QIAcuity One 5-plex Digital PCR System (Qiagen, Hilden, Germany). Thermal cycling conditions included reverse transcription at 50°C for 30 minutes, initial denaturation at 95°C for 2 minutes, followed by 45 amplification cycles of 95°C for 10 seconds and 55°C for 30 seconds. The system operated in absolute quantification mode, and data were analyzed using QIAcuity Software Suite v2.2. Viral concentrations were reported as gene copies per milliliter (GC/mL) of wastewater, calculated using the following equation:


$$\frac{GC}{mL}=\frac{\left(\frac{\text{Copies in reaction}}{\text{Template volume }(\mu \text{L})}\right)\times \text{Elution volume }(\mu \text{L})}{\text{Sample volume }(\text{mL})}$$

where “Copies in reaction” is the absolute copy number output, “Template volume” is the RNA input volume (µL), “Elution volume” is the total volume used for RNA elution (µL), and “Sample volume” is the squeezed wastewater from tampons (mL) used per replicate for RNA extraction.

### Library preparation and sequencing for wastewater samples

RNAs extracted from six wastewater samples were used for targeted sequencing of respiratory viruses. These samples were selected based on positive SARS-CoV-2 detection by RT-dPCR: four consecutive samples collected from 7/23/2024 to 8/13/2024, coinciding with the COVID-19 outbreak period at the nursing home, and two consecutive samples collected from 9/17/2024 to 9/25/2024, representing a second detection event 1 month later. Library preparation was performed using the Illumina Respiratory Virus Enrichment Kit (RVEK; Illumina, CA, USA), which utilizes hybridization-based targeted enrichment sequencing targeting 40 different respiratory viruses, including SARS-CoV-2, influenza A, influenza B, and RSV. First, RNA concentration for each sample was measured using the Qubit 4 Fluorometer with the RNA Broad Range (BR) Assay Kit (Thermo Fisher Scientific, MA, USA) to ensure accurate input for library preparation. cDNA was then synthesized from RNA, followed by 24-hour hybridization-based enrichment of viral targets and library indexing using Illumina DNA/RNA Unique Dual Indexes (Illumina, CA, USA). Three wastewater samples were pooled per sequencing library, resulting in two pools (*n* = 3 per pool). Each pooled library was diluted to a final loading concentration of 8 pM, and the sequencing was carried out on the Illumina MiSeq platform using the MiSeq V2 Reagent Kit (2 × 150 cycles), according to the manufacturer’s instructions.

### Nasopharyngeal swab for clinical testing

The wastewater surveillance team reported weekly wastewater SARS-CoV-2 results to the nursing home, which then informed the epidemiologists of its residents’ clinical symptoms and any control responses taken. When SARS-CoV-2 was first detected at low concentrations on 7/23/2024, and again at similarly low concentrations the following week (7/30/2024), the wastewater team promptly notified both the epidemiologists and nursing home staff. The subsequent week (8/6/2024) showed a significant increase in viral concentration, which preceded a rise in COVID-19 cases among residents using at-home rapid antigen testing kits. This escalating signal prompted action: nasopharyngeal (NP) swab testing was approved, and kits were provided to the nursing home on 8/13/2024 for staff-collected sampling. NP swab samples were collected on two separate dates, 8/14/2024 and 8/20/2024, and the samples were received at the laboratory on the following days, 8/15/2024 and 8/21/2024, respectively. The NP swabs were performed in accordance with the Centers for Disease Control and Prevention (CDC)’s nasal mid-turbinate (NMT) specimen collection protocol (33). Briefly, this method involves inserting a swab approximately 1–1.5 inches into each nostril (until resistance is met), rotating it several times against the nasal wall, and placing it into a sterile transport tube. The collected NP swabs were stored on ice until epidemiologists retrieved the samples and transported them to the laboratory with ice coolers for RNA extraction.

### RNA extraction from clinical samples and RT-qPCR

NP swab samples collected from nursing home residents were processed for SARS-CoV-2 detection. Viral RNA was extracted using the Thermo Scientific KingFisher Flex Purification System in a 96 deep-well plate format with the MagMAX Viral/Pathogen II Nucleic Acid Isolation Kit (Thermo Fisher Scientific, MA, USA). The extraction procedure followed the manufacturer’s protocol, which is optimized for high-throughput automated purification of viral RNA from swab transport media. SARS-CoV-2-positive samples were initially screened using real-time reverse transcription PCR (RT-qPCR) with the Applied Biosystems TaqPath COVID-19 Multiplex Kit (Thermo Fisher Scientific, MA, USA), which targets the N gene, surface glycoprotein (S) gene, and open reading frame (ORF)1ab genes of the SARS-CoV-2 genome on an Applied Biosystems 7500DX Real-Time PCR instrument (Thermo Fisher Scientific, MA, USA). However, the SARS-CoV-2 lineage detected in the NP swab samples exhibited S gene dropout, possibly due to mutations in the S gene primer/probe binding region. Consequently, only samples with strong amplification of both the N and ORF1ab genes with Ct values ≤26 were selected for downstream sequencing for sufficient viral RNA input and comprehensive genome coverage.

### Library preparation and sequencing of clinical samples

A total of 15 SARS-CoV-2-positive clinical samples were prepared for sequencing, grouped according to similar RT-qPCR Ct values to ensure even representation. For each sample, 6 µL of RNA was subjected to overnight hybridization using the Molecular Loop Viral RNA Target Capture Kit (Molecular Loop Biosciences, MA, USA). This step enabled targeted capture through molecular inversion probes (MIPs), followed by a gap-fill reaction to complete probe circularization. Post-capture, a series of clean-up steps was performed to remove unincorporated probes, enzymatically release probes from the target RNA, attach unique index barcodes using Illumina DNA/RNA Unique Dual Indexes (Illumina, CA, USA), and conduct a limited-cycle PCR amplification to enrich targeted sequences. Sample concentrations were first quantified using the Qubit Flex Fluorometer with the dsDNA BR Assay Kit (Thermo Fisher Scientific, MA, USA). Libraries were then pooled and normalized based on DNA concentration, followed by purification using AMPure XP Beads (Beckman Coulter, CA, USA). The final pool was quantified using the Qubit 4 Fluorometer with the dsDNA High-Sensitivity (HS) Assay Kit (Thermo Fisher Scientific, MA, USA) and diluted to 4 nM using the supplied resuspension buffer. For sequencing, the pooled library was diluted to a final loading concentration of 10 pM, and sequencing was conducted using the MiSeq Reagent Micro Kit v2 (2  ×  150 cycles) on an Illumina MiSeq instrument (Illumina, CA, USA).

### Sequencing data analysis

Sequencing data from both wastewater and clinical samples were analyzed using CLC Genomics Workbench (version 25.0.1; Qiagen, Hilden, Germany). Raw FASTQ files were downloaded from Illumina BaseSpace (Illumina, CA, USA) and either imported directly (wastewater) or after adapter trimming (clinical) into the software. Clinical reads were trimmed of MIP adapter arms using Cutadapt (34) and Ngmerge (35) prior to import into CLC. Quality control was performed using the built-in CLC trimming and filtering tools to remove low-quality bases (quality score less than 10) and adapter sequences (wastewater). Reads were then mapped to the SARS-CoV-2 reference genome (SARS-CoV-2/human/USA/UT-UPHL-231213971510/2023; GenBank accession: OR983129.1; total length: 29,763 bp) using the CLC read mapper with default parameters optimized for viral genomes. Consensus sequences were generated from the mapped reads, and low-coverage regions (<30× ) were masked. Detailed raw sequencing metrics are provided in Table S1.

Variant calling was performed to identify single-nucleotide variants (SNVs), insertions, and deletions relative to the reference genome. Consensus genomes were exported as FASTA files and further evaluated for lineage and mutation profiling and phylogenetic tree generation using the Nextclade web tool (version 3.15.0; https://clades.nextstrain.org/). Clade assignment and mutation analysis were conducted using the SARS-CoV-2 reference genome Wuhan-Hu-1/2019 (GenBank accession: NC_045512.2; total length: 29,903 bp). The phylogenetic tree was constructed using the SARS-CoV-2 reference genome SARS-CoV-2/human/USA/CA-CDPH-500140229/2024 (GenBank accession: PQ134785.1). The consensus sequences were analyzed using NCBI BLAST against the NCBI Virus Database (https://www.ncbi.nlm.nih.gov/labs/virus/vssi/#/) to assess their similarity to other isolated or detected SARS-CoV-2 sequences. Other respiratory viruses targeted by the targeted enrichment sequencing kit were identified from the wastewater samples using the DRAGEN Microbial Enrichment Plus pipeline 1.1.0 (Illumina, CA, USA).

## Data Availability

Raw sequencing data are available in the NCBI Sequence Read Archive (SRA) under accession numbers SRR35771564 to SRR35771577 for clinical samples and SRR35771773 to SRR35771778 for wastewater samples, BioSample accession numbers SAMN52637126 to SAMN52637144 for clinical samples and SAMN52640139 to SAMN52640144 for wastewater samples, all under BioProject number PRINJA1344338.

## SUPPLEMENTAL MATERIAL

Supplemental material aem.00757-26-s0001.docx Fig. S1 to S3; Tables S1 to S3.

ASM does not own the copyrights to Supplemental Material that may be linked to, or accessed through, an article. The authors have granted ASM a non-exclusive, world-wide license to publish the Supplemental Material files. Please contact the corresponding author directly for reuse.
