# Supplementary material for: Detecting and supporting COVID-19 outbreak response in a small-scale nursing home through passive wastewater surveillance of SARS-CoV-2
Source: Appl Environ Microbiol. 2026 Jul 23;92(8):e00757-26. doi: 10.1128/aem.00757-26 (PMC13488356; doi:10.1128/aem.00757-26)
Supplement: Supplementary file 1 [file aem.00757-26-s0001.docx]

**Supplementary Information of**

**Detecting and Supporting COVID-19 Outbreak Response in a Low-Population Nursing Home Through Passive Wastewater Surveillance of SARS-CoV-2**

Min Ki Jeon^1, 2^, Doris Yoong Wen Di^3^, Maria Steadmon^3^, Stella Maris Remigio^3^, Pamela O’Brien^3^, Daniel P. Strange^3^, Vila Chanthasouvanh^4^, Dayna Ornellas^3^, Edward Desmond^3^, and Tao Yan^1, 2*^

^1^ Water Resources Research Center, University of Hawai‘i at Mānoa, Honolulu, HI 96822, United States

^2^ Department of Civil, Environmental and Construction Engineering, University of Hawai‘i at Mānoa, Honolulu, HI 96822, United States

^3^ State Laboratories Division, State of Hawai‘i Department of Health, Pearl City, HI 96782, United States

^4^ Disease Outbreak Control Division, State of Hawai‘i Department of Health, Honolulu, HI 96813, United States

*Corresponding author: Tao Yan, University of Hawai‘i at Mānoa, Department of Civil, Environmental and Construction Engineering, T706 Biomedical Sciences Building, 1960 East-West Rd, Honolulu, HI 96822, United States. Phone: 808-956-6024. Fax: 808-956-5014. E-mail: [taoyan@hawaii.edu](mailto:taoyan@hawaii.edu)

**Keywords:** Passive wastewater sampling, respiratory virus, nursing home, low-population, sequencing

**Figure S1.** Detection of pepper mild mottle virus (PMMoV; turquoise circle) and bovine coronavirus (BCoV; brown triangle) by RT-dPCR in wastewater sampled with a tampon-based passive sampler from a small nursing home. The upper *x*-axis shows sampling weeks, and the lower *x*-axis shows sampling dates. The error bars indicate standard deviation.

**Figure S2.** Correlation plot between Ct values of the N gene and ORF1ab gene of SARS-CoV-2 detected by RT-qPCR in clinical samples.

**Figure S3.** Mapped reads per nucleotide position across the full-length SARS-CoV-2 genome (29,903 bp) from wastewater and clinical samples, showing paired (navy), forward unpaired (green), and reverse unpaired (red) reads.

**Table S1.** Detailed information on raw sequencing data used for mapping with CLC genomics

| **Sample type** | **Sample** | **Number of reads*** | **Average length (bp)** | **Total reads after trim** | **Trimmed reads** | **Trimmed (broken pairs)** | **Percentage trimmed (%)** | **Average length after trim (bp)** | **Mapped target reads (% of total reads)** | **Not mapped reads (% of total reads)** |
| --- | --- | --- | --- | --- | --- | --- | --- | --- | --- | --- |
| Wastewater | W1 | 4,050,008 | 108.4 | 3,541,572 | 3,535,324 | 6,248 | 87.5 | 117.9 | 661 (0.0%) | 3,534,645 (100%) |
|  | W2 | 4,032,170 | 122.3 | 3,774,483 | 3,770,162 | 4,321 | 93.6 | 127.6 | 2,388 (0.1%) | 3,767,762 (99.9%) |
|  | W3 | 3,564,306 | 123.2 | 3,355,177 | 3,351,826 | 3,351 | 94.1 | 128.1 | 46,963 (1.4%) | 3,304,847 (98.6%) |
|  | W4 | 8,214,752 | 130.3 | 7,968,023 | 7,953,518 | 14,505 | 97.0 | 132.7 | 539,135 (6.8%) | 7,414,380 (93.2%) |
|  | W5 | 10,164,482 | 130.1 | 9,818,035 | 9,802,806 | 15,229 | 96.6 | 132.9 | 474 (0.0%) | 9,802,308 (100%) |
|  | W6 | 12,121,426 | 127.4 | 11,611,478 | 11,592,786 | 18,692 | 95.8 | 130.8 | 46 (0.0%) | 11,592,721 (100%) |
| Clinical | C1 | 193,000 | 120.7 | 192,992 | 192,996 | 4 | 100.0 | 120.3 | 152,676 (79.1%) | 40,316 (20.9%) |
|  | C2 | 365,058 | 120.7 | 365,050 | 365,042 | 8 | 100.0 | 120.3 | 237,270 (65.0%) | 127,772 (35.0%) |
|  | C3 | 358,748 | 120.8 | 358,739 | 358,730 | 9 | 100.0 | 120.3 | 181,980 (50.7%) | 176,750 (49.3%) |
|  | C4 | 237,556 | 120.9 | 237,545 | 237,534 | 11 | 100.0 | 120.2 | 58,768 (24.7%) | 178,766 (75.3%) |
|  | C5 | 741,870 | 120.9 | 741,849 | 741,828 | 21 | 100.0 | 120.3 | 183,256 (24.7%) | 558,572 (75.3%) |
|  | C6 | 410,076 | 121.0 | 410,056 | 410,036 | 20 | 100.0 | 120.4 | 5,215 (1.3%) | 404,821 (98.7%) |
|  | C7 | 366,646 | 121.0 | 366,636 | 366,626 | 10 | 100.0 | 120.3 | 2,172 (0.6%) | 364,454 (99.4%) |
|  | C8 | 509,168 | 121.0 | 509,139 | 509,110 | 29 | 100.0 | 120.1 | 73,786 (14.5%) | 435,324 (85.5%) |
|  | C9 | 570,300 | 120.9 | 570,277 | 570,254 | 23 | 100.0 | 120.4 | 171,809 (30.1%) | 398,445 (69.9%) |
|  | C10 | 370,468 | 121.0 | 370,454 | 370,440 | 14 | 100.0 | 120.3 | 8,947 (2.4%) | 361,493 (97.6%) |
|  | C11 | 437,326 | 121.0 | 437,310 | 437,294 | 16 | 100.0 | 120.1 | 14,403 (3.3%) | 422,891 (96.7%) |
|  | C12 | 497,338 | 121.0 | 497,325 | 497,312 | 13 | 100.0 | 120.3 | 51,037 (10.3%) | 446,275 (89.7%) |
|  | C13 | 463,702 | 121.0 | 463,686 | 463,670 | 16 | 100.0 | 120.3 | 9,752 (2.1%) | 453,918 (97.9%) |
|  | C15 | 397,556 | 121.0 | 397,540 | 397,524 | 16 | 100.0 | 120.4 | 65,374 (16.4%) | 332,150 (83.6%) |

* Clinical reads were trimmed of MIP adapter arms using Cutadapt [1] and NGmerge [2] prior to import into CLC.

**Table S2.** Detailed information regarding the consensus sequences of detected viruses from wastewater and clinical samples using Nextclade

| **Sample type** | **Sample** | **Detected virus** | **Closest lineage** | **Segment*** | **Aligned reference GenBank accession** | **Reference length (bp)** | **Total assembled length (bp)** | **Aligned length (bp)** | **Number of Ns** | **Coverage** | **Total aligned read count** | **Median read depth** |
| --- | --- | --- | --- | --- | --- | --- | --- | --- | --- | --- | --- | --- |
| Wastewater | W1 | SARS-CoV-2 | Omicron (BA.1.1) | - | NC_045512.2 | 29,903 | 29,763 | 948 | 28,815 | 3.2% | 278 | 0 |
|  | W2 | SARS-CoV-2 | Omicron (MK.1) | - | NC_045512.2 | 29,903 | 29,762 | 6,383 | 23,379 | 21.3% | 2,115 | 13.0 |
|  |  | Influenza A | H3N2 | 1 (PB2) | KY926316.1 | 2,341 | 2,341 | 279 | 2,062 | 11.9% | 16 | 0 |
|  |  |  |  | 4 (HA) | KY926260.1 | 1,762 | 1,762 | 384 | 1,378 | 21.8% | 161 | 0 |
|  |  |  |  | 5 (NP) | KY925574.1 | 1,566 | 1,566 | 64 | 1,502 | 4.1% | 2 | 0 |
|  |  |  |  | 6 (NA) | KY925971.1 | 1,467 | 1,467 | 42 | 1,425 | 2.9% | 22 | 0 |
|  |  |  |  | 7 (M) | KY925498.1 | 1,027 | 1,027 | 142 | 885 | 13.8% | 90 | 0 |
|  | W3 | SARS-CoV-2 | Omicron (LB.1.5) | - | NC_045512.2 | 29,903 | 29,760 | 29,285 | 475 | 97.9% | 47,884 | 151.3 |
|  | W4 | SARS-CoV-2 | Omicron (LB.1.5) | - | NC_045512.2 | 29,903 | 29,760 | 29,739 | 21 | 99.5% | 554,563 | 1,602.1 |
|  |  | Influenza A | H1N1 | 1 (PB2) | NC_026438.1 | 2,280 | 2,280 | 151 | 2,129 | 6.6% | 56 | 0 |
|  |  |  |  | 3 (PA) | NC_026437.1 | 2,151 | 2,151 | 481 | 1,670 | 22.4% | 104 | 0 |
|  |  |  |  | 5 (NP) | NC_026436.1 | 1,497 | 1,497 | 83 | 1,414 | 5.5% | 18 | 0 |
|  |  |  |  | 8 (NEP/  NS1) | NC_026432.1 | 863 | 863 | 46 | 817 | 5.3% | 12 | 0 |
| Clinical | C1 | SARS-CoV-2 | Omicron (LB.1.5) | - | NC_045512.2 | 29,903 | 29,700 | 29,700 | 0 | 99.3% | 159,008 | 732.3 |
|  | C2 | SARS-CoV-2 | Omicron (LB.1.5) | - | NC_045512.2 | 29,903 | 29,760 | 29,700 | 60 | 99.3% | 245,277 | 997.1 |
|  | C3 | SARS-CoV-2 | Omicron (LB.1.5) | - | NC_045512.2 | 29,903 | 29,760 | 29,700 | 60 | 99.3% | 187,113 | 670.8 |
|  | C4 | SARS-CoV-2 | Omicron (LB.1.5) | - | NC_045512.2 | 29,903 | 29,760 | 29,690 | 70 | 99.3% | 59,085 | 198.8 |
|  | C5 | SARS-CoV-2 | Omicron (LB.1.5) | - | NC_045512.2 | 29,903 | 29,760 | 29,700 | 60 | 99.3% | 192,165 | 1098 |
|  | C6 | SARS-CoV-2 | Omicron (LB.1.5) | - | NC_045512.2 | 29,903 | 29,760 | 22,722 | 7,038 | 76.0% | 4,649 | 20.0 |
|  | C7 | SARS-CoV-2 | Omicron (KP.2.3) | - | NC_045512.2 | 29,903 | 29,763 | 3,634 | 26,129 | 12.2% | 1,434 | 4.6 |
|  | C8 | SARS-CoV-2 | Omicron (LB.1.5) | - | NC_045512.2 | 29,903 | 29,760 | 29,700 | 60 | 99.3% | 73,480 | 346.3 |
|  | C9 | SARS-CoV-2 | Omicron (LB.1.5) | - | NC_045512.2 | 29,903 | 29,760 | 29,700 | 60 | 99.3% | 175,108 | 626.7 |
|  | C10 | SARS-CoV-2 | Omicron (LB.1.5) | - | NC_045512.2 | 29,903 | 29,760 | 28,192 | 1,568 | 94.3% | 5,398 | 20.5 |
|  | C11 | SARS-CoV-2 | Omicron (LB.1.5) | - | NC_045512.2 | 29,903 | 29,760 | 29,549 | 211 | 98.8% | 12,695 | 44.0 |
|  | C12 | SARS-CoV-2 | Omicron (LB.1.5) | - | NC_045512.2 | 29,903 | 29,760 | 29,683 | 77 | 99.3% | 48,924 | 182.4 |
|  | C13 | SARS-CoV-2 | Omicron (LB.1.5) | - | NC_045512.2 | 29,903 | 29,760 | 29,020 | 740 | 97.0% | 6,287 | 22.4 |
|  | C15 | SARS-CoV-2 | Omicron (LB.1.5) | - | NC_045512.2 | 29,903 | 29,760 | 29,690 | 70 | 99.3% | 59,475 | 316.9 |

* Segments of the influenza virus genome. PB2: basic polymerase 2; PA: polymerase acidic; HA: haemagglutinin; NP: nucleocapsid protein; NA: neuraminidase; M: matrix protein; NEP/NS1: nuclear export protein/nonstructural protein 1.

**Table S3.** Timeline of wastewater SARS-CoV-2 surveillance, clinical case counts, and infection control actions during the COVID-19 outbreak at the nursing home (July–August 2024)

| **Surveillance Week** | **Date** | **WW Sample ID** | **SARS-CoV-2 Concentration (GC/mL)** | **COVID-19 Cases (Cumulative)** | **Key Events and Actions** |
| --- | --- | --- | --- | --- | --- |
| Week 44 | 7/23/2024 | W1 | 57.6 ± 7.5 | 0 | Wastewater team notified nursing home facility administrator on 7/26/2024. One staff member had been out with COVID-19 the previous week. |
| Week 45 | 7/29/2024 | – | – | 0 | Facility administrator reported no residents exhibited respiratory symptoms or tested positive by at-home rapid antigen test. A newly hired activities assistant began working on 7/29/2024. A family member of a resident visited the facility daily from 7/28/2024 to 8/1/2024. |
| Week 45 | 7/30/2024 | W2 | 7.5 ± 2.8 | 0 | No residents exhibited respiratory symptoms or tested positive by at-home rapid antigen test. |
| Week 45 | 8/1/2024 | – | – | 0 | Newly hired activities assistant tested positive for COVID-19 and remained at home until testing negative. |
| Week 46 | 8/6/2024 | W3 | 1.3 × 10^4^ ± 2.1 × 10^2^ | 0 | Wastewater team reported >1,700-fold increase in SARS-CoV-2 concentration on 8/8/2024. Activities assistant tested negative and returned to work on 8/6/2024. No staff or residents had tested positive or shown symptoms at this time. |
| Week 46 | 8/8/2024 | – | – | 0 | Family member who visited daily from 7/28–8/1/2024 tested positive for COVID-19 by at-home rapid antigen test. |
| Week 46 | 8/10/2024 | – | – | 6 | Six residents confirmed positive for COVID-19 by at-home rapid antigen test (evening). Residents began Paxlovid treatment and placed in isolation. |
| Week 46 | 8/12/2024 | – | – | 11 | Five additional residents confirmed positive. Facility implemented cohorting for activities and meals, visitor restrictions, enhanced cleaning, and suspended new admissions. Staff equipped with full PPE (N95 respirators, gowns, gloves, face shields). |
| Week 47 | 8/13/2024 | W4 | 3.6 × 10^4^ ± 4.6 × 10^4^ | 11 | NP swab kits provided to nursing home. |
| Week 47 | 8/14/2024 | – | – | 14 | 14 residents confirmed positive by NP swab RT-qPCR. All staff tested negative and remained asymptomatic. |
| Week 48 | 8/20/2024 | – | – | 15 | One additional resident confirmed positive by both at-home rapid antigen test and NP swab RT-qPCR. No further cases reported after this date. |

WW: wastewater; GC/mL: gene copies per milliliter; NP: nasopharyngeal; PPE: personal protective equipment; –: not applicable.

Wastewater concentrations are reported as mean ± standard deviation. COVID-19 cases reflect confirmed positive results from at-home rapid antigen tests except where noted as NP swab RT-qPCR.

**References**

1.	Martin, M., *Cutadapt removes adapter sequences from high-throughput sequencing reads.* EMBnet. journal, 2011. **17**(1): p. 10-12.

2.	Gaspar, J.M., *NGmerge: merging paired-end reads via novel empirically-derived models of sequencing errors.* BMC bioinformatics, 2018. **19**(1): p. 536.
